# Mitigation of Drought Stress for Quinoa (*Chenopodium quinoa* Willd.) Varieties Using Woodchip Biochar-Amended Soil

**DOI:** 10.3390/plants13162279

**Published:** 2024-08-15

**Authors:** Muhammad Zubair Akram, Anna Rita Rivelli, Angela Libutti, Fulai Liu, Christian Andreasen

**Affiliations:** 1 Ph.D. Program in Agricultural, Forest and Food Sciences, University of Basilicata, Via dell’Ateneo Lucano 10, 85100 Potenza, Italy; muhammadzubair.akram@unibas.it; 2 School of Agricultural, Forest, Food and Environmental Sciences, University of Basilicata, Via dell’Ateneo Lucano 10, 85100 Potenza, Italy; annarita.rivelli@unibas.it; 3 Department of Plant and Environmental Sciences, University of Copenhagen, Højbakkegaard Allé 13, 2630 Taastrup, Denmark; fl@plen.ku.dk; 4 Department of Agricultural Sciences, Food, Natural Resources and Engineering (DAFNE), University of Foggia, Via Napoli 25, 71122 Foggia, Italy; angela.libutti@unifg.it

**Keywords:** drought resistance, drought tolerance, physiological parameters, morphological attributes, root traits, soil amendments

## Abstract

Drought stress deteriorates agro-ecosystems and poses a significant threat to crop productivity and food security. Soil amended with biochar has been suggested to mitigate water stress, but there is limited knowledge about how biochar affects the physiology and vegetative growth of quinoa plants under soil water deficits. We grew three quinoa (*Chenopodium quinoa* Willd.) varieties, Titicaca (V1), Quipu (V2), and UAFQ7 (V3) in sandy loam soil without (B0) and with 2% woodchip biochar (B2) under drought conditions. The drought resulted in significant growth differences between the varieties. V3 performed vegetatively better, producing 46% more leaves, 28% more branches, and 25% more leaf area than the other two varieties. Conversely, V2 displayed significantly higher yield-contributing traits, with 16% increment in panicle length and 50% more subpanicles compared to the other varieties. Woodchip biochar application significantly enhanced the root development (i.e., root biomass, length, surface, and projected area) and plant growth (i.e., plant height, leaf area, and absolute growth rate). Biochar significantly enhanced root growth, especially fresh and dry weights, by 122% and 127%, respectively. However, biochar application may lead to a trade-off between vegetative growth and panicle development under drought stress as shown for V3 grown in soil with woodchip biochar. However, V3B2 produced longer roots and more biomass. Collectively, we suggest exploring the effects of woodchip biochar addition to the soil on the varietal physiological responses such as stomatal regulations and mechanisms behind the increased quinoa yield under water stress conditions.

## 1. Introduction

Quinoa (*Chenopodium quinoa* Willd.) belongs to the Amaranthaceae family [1]. It is an annual C_3_ crop originally from the South American highlands known for its tolerance to adverse abiotic stresses, particularly salinity and drought [2]. It demonstrates remarkable adaptability to grow in poor soils and under extreme climatic conditions [3]. Quinoa’s drought tolerance is caused by its deep and extensive root system, white reflective and hygroscopic papillae on the leaf surface [4], leaf area reduction by shedding leaves to reduce water loss through transpiration, presence of special vesicular glands (i.e., small thick-walled cells used for water storage reservoirs maintaining turgor during water limitations [5]), and stomatal closure [6]. Jensen et al. [7] revealed that the leaf water potential threshold for stomatal closure in quinoa varies between cultivars, occurring at −1.2 to −1.6 MPa. Quinoa’s tolerance to drought is also attributed to its phenotypic plasticity, tissue elasticity, and inherently low osmotic potential [7]. This phenotypic plasticity, highlighted by Stanschewski et al. [8], underscores the importance of exploring quinoa germplasm from diverse geographical locations. The vast genetic diversity within quinoa germplasm includes genes conferring resistance to both biotic and abiotic stresses [9], suggesting its crucial role in quinoa’s ability to adapt to various environmental challenges.

Inter-varietal variation in drought tolerance mechanisms necessitates targeted screening of quinoa germplasm for specific water deficit conditions [10]. Earlier studies have shown that significant variations were evident between five quinoa ecotypes in drought tolerance mechanisms [11]. The ecotype Salares performed better under drought stress than other ecotypes through extensive root systems, low osmotic potential, epidermal bladder cells and stomatal control [4]. Notable varieties from this ecotype are UAFQ7 [2] and Quipu [12], the first approved quinoa varieties in Pakistan and Italy, respectively. Akram et al. [2] studied 13 superior genotypes screened from 117 imported quinoa lines from the United States Department of Agriculture (USDA) [10] and showed that UAFQ7 has longer roots and higher yield than other genotypes. Similarly, Valley and Altiplano ecotypes are considered short-duration and fast-growing varieties, like Titicaca, that showed drought escape mechanisms by completing their life cycle earlier to avoid the drought effects at later stages. However, it was more susceptible to water deficits under prolonged drought conditions [13].

Application of organic amendments, including compost, manure, and biochar, can significantly mitigate the adverse effects of water stress on plant growth [14]. They improve soil structure, water retention, and nutrient availability [15]. Among these, biochar is particularly advantageous due to its exceptional water-holding capacity, nutrient adsorption, and ability to stimulate beneficial soil microbial communities, and thereby enhance plant growth under water stress conditions [16]. Biochar, a carbon-rich byproduct of pyrolysis [17], offers a promising approach to mitigating climate change and enhancing agricultural productivity under drought stress. Several studies have documented its positive effects on plant growth [18,19], yield [20], nutrient uptake [21], and plant physiological properties [22] under drought. Specifically for quinoa, biochar application has been shown to enhance both vegetative [23] and reproductive growth [18] under drought stress through changes in soil physicochemical and biological properties [24]. Studies have documented significant improvement in soil structure, water-holding capacity, and other physicochemical properties under drought conditions after biochar application [25]. The improved soil environment enhances plant biomass and nutrient uptake [18]. Furthermore, biochar application positively influences plant physiological attributes under drought stress, such as increased photosynthetic rate, stomatal conductance, and plant water status in quinoa [26]. Biochar’s porous structure retains water and may enhance root proliferation and growth. Thus, an improved root system could maintain leaf turgor pressure, stomatal conductance and photosynthesis during drought stress by facilitating increased water and nutrient uptake [27]. In biochar-amended soil, Kammann et al. [18] observed better quinoa growth and water-use efficiency under drought conditions.

Apart from its advantages, biochar can adversely affect plant growth by immobilizing essential nutrients through altering soil pH [28]. High application rates can create physical barriers, hindering root development due to the highly porous nature of the biochar which accelerates the loss of water and nutrients from the soil [29]. The influence of biochar application on quinoa’s physiological response to drought stress throughout vegetative development remains understudied [30]. While previous work has shown that biochar can enhance the growth of the drought-sensitive quinoa cultivar Titicaca [23], the effect under prolonged water deficits remains unknown [19].

With the significant phenotypic diversity within quinoa germplasm in mind, this study aimed to contribute substantially to our understanding of drought tolerance mechanisms of quinoa varieties under biochar application. We studied the impact of woodchip biochar application at a rate of 2% in the soil to enhance drought tolerance among different quinoa varieties grown under water stress conditions. We utilized morphological and physiological analyses to evaluate the impact of successive water stress cycles on quinoa growth and physiology to elucidate the interaction between drought stress and biochar application, mainly focusing on their combined effects during the vegetative growth stage. We hypothesized that application of woodchip biochar to the soil helps the quinoa varieties to avoid the negative impact of water stress conditions occurring during the vegetative growing cycle of the plant.

## 2. Results

### 2.1. Plant Growth and Biomass during the Experiment

**Figure 1:**
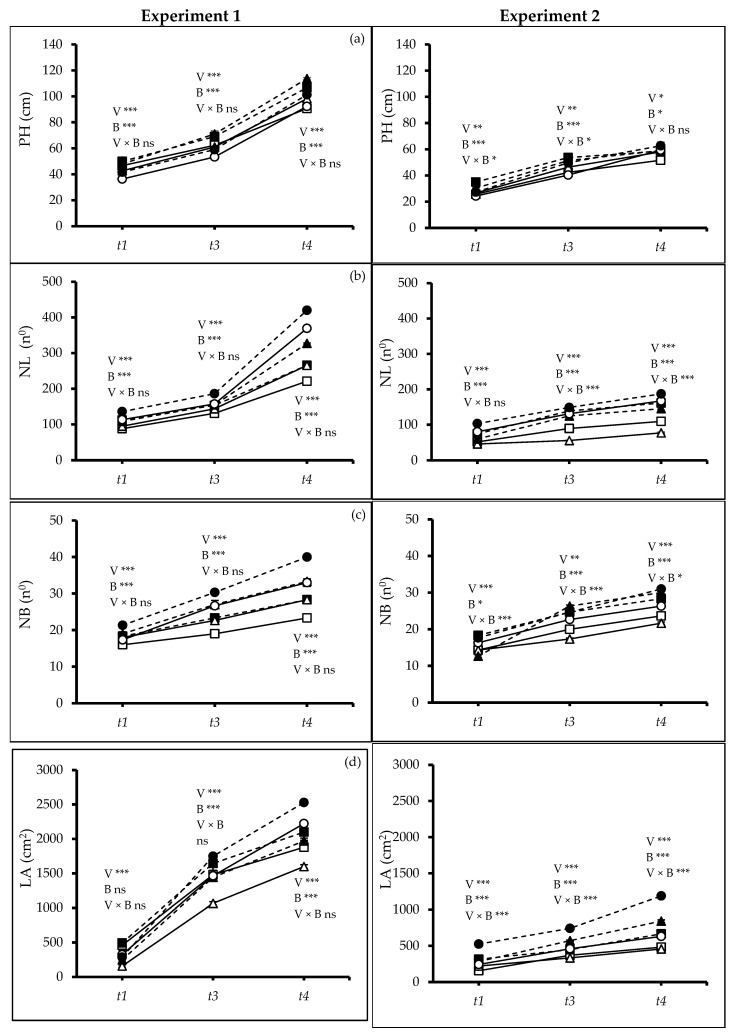
(**a**) Plant height (PH), (**b**) number of leaves (NL), (**c**) number of branches (NB), and (**d**) leaf area (LA) of quinoa (*Chenopodium quinoa* Willd.) varieties treated without and with woodchip biochar. Values are means (*n* = 3) ± S.E. In each graph, different stars above and below the lines indicate significant differences among treatments (*p* ≤ 0.05, Tukey’s test). F-test significant at *—(*p* ≤ 0.05), **—(*p* ≤ 0.01), ***—(*p* ≤ 0.001). *t_1_*—start of drought, *t_3_*—end of 2nd drought cycle and *t_4_*—end of the experiment. White squares: quinoa variety 1 (V1) without biochar. Black squares: quinoa variety 1 (V1) with biochar; white triangles: quinoa variety 2 (V2) without biochar; black triangles: quinoa variety 2 (V2) with biochar; white circles: quinoa variety 3 (V3) without biochar; black circles: quinoa variety 3 (V3) with biochar; solid lines—(B0) no biochar, dash lines—(B2) 2% biochar.

At all three harvests (*t_1_*, *t_3_* and *t_4_*) in both experiments, there were significant differences between the varieties’ response to biochar (Exp. 1: *t_1_*: *p* ≤ 0.0008; *t_3_*: *p* ≤ 0.0000; *t*_4_: *p* ≤ 0.0066; Exp. 2: *t_1_*: *p* ≤ 0.0042; *t_3_*: *p* ≤ 0.0024; *t*_4_: *p* ≤ 0.0320) in height (PH). Plants grown in biochar-amended soil were taller (Exp. 1: *t_1_*: *p* ≤ 0.0055; *t_3_*, *t*_4_: *p* ≤ 0.0000; Exp. 2: *t_1_*, *t_3_*: *p* ≤ 0.0000; *t_4_*: *p* ≤ 0.0329), but there was no significant interaction between varieties and soil type. In both experiments, V1 was the tallest at *t_1_*, while V2 was the tallest at the final harvest (*t*_4_) (Figure 1a). Contrarily, in Exp. 2, an interaction was observed for plant height at *t_1_* and *t_3_* (*t_1_*, *p* ≤ 0.0227; *t_3_*, *p* ≤ 0.0109). In particular, V1 grown with biochar attained the greatest height at both harvests. Plants grown in soil with biochar achieved higher numbers of leaves (NL), branches (NB), and larger leaf areas (LA). Similarly, an interaction was recorded for the number of leaves and branches at *t_3_* and *t_4_* for Exp. 2 (*t_3_*, *t_4_*: *p* ≤ 0.0000). At both times, V3 showed a positive response to biochar possessing a higher number of leaves and branches.

In both experiments, at all three harvest times, V3 produced the highest number of leaves (Exps. 1 and 2: *t_1_*, *t_3_*, *t*_4_: *p* ≤ 0.0000), branches (Exp. 1: *t*_1_: *p* ≤ 0.0008; *t_3_*, *t*_4_: *p* ≤ 0.0000; Exp. 2: *t_1_*, *t*_4_: *p* ≤ 0.0000; *t_3_*: *p* ≤ 0.0013), and leaf areas (Exps. 1 and 2: *t_1_*, *t_3_*, *t*_4_: *p* ≤ 0.0000). All varieties grown in soil with biochar had significantly higher numbers of leaves, branches, and leaf areas at all harvests (Figure 1b–d). A significant two-factor interaction was observed in Exp. 2 at *t_1_*, *t_3_* and *t_4_* (*t_1_*, *t_3_*, *t_4_*: *p* ≤ 0.0000). Consistent with the number of leaves and branches, V3 showed the positive effect of biochar addition by always producing bigger leaf areas. Importantly, at *t_4_* biochar treatment enhances the number of leaves by 18% and 40% in Exp. 1 and 2, respectively. Similarly, plants grown with biochar produced bigger leaf areas than non-amended ones by 20% and 60% in Exp 1. and 2, respectively.

**Figure 2:**
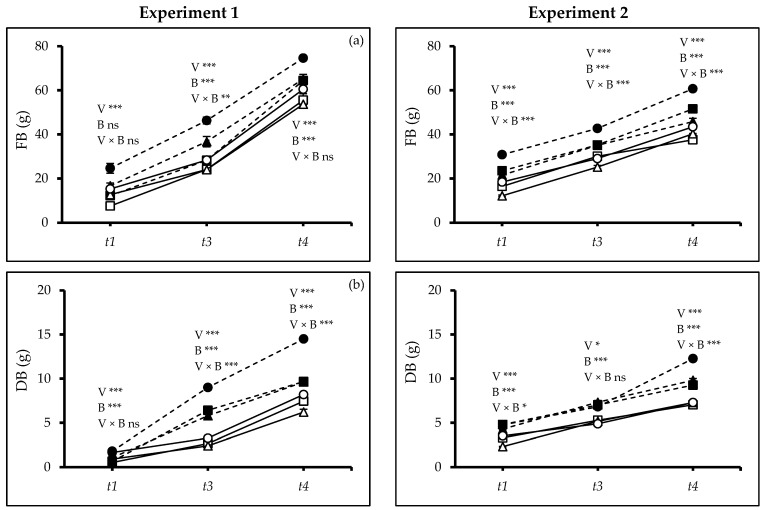
(**a**) Fresh biomass (FB), and (**b**) dry biomass (DB) of quinoa (*Chenopodium quinoa* Willd.) varieties treated without and with woodchip biochar. Values are means (*n* = 3) ± S.E. In each graph, different stars above and below the lines indicate significant differences among treatments (*p* ≤ 0.05, Tukey’s test). F-test significant at *—(*p* ≤ 0.05), **—(*p* ≤ 0.01), ***—(*p* ≤ *0*.001). *t_1_*—start of drought, *t_3_*—end of 2nd drought cycle and *t_4_*—end of the experiment. White squares: quinoa variety 1 (V1) without biochar. Black squares: quinoa variety 1 (V1) with biochar; white triangles: quinoa variety 2 (V2) without biochar; black triangles: quinoa variety 2 (V2) with biochar; white circles: quinoa variety 3 (V3) without biochar; black circles: quinoa variety 3 (V3) with biochar; solid lines—(B0) no biochar, dash lines—(B2) 2% biochar.

In both experiments, the fresh biomass (FB) varied between varieties (Exp. 1: *t_1_*, *t_3_*: *p* ≤ 0.0000, *t_4_*: *p* ≤ 0.0008; Exp. 2: *t_1_*, *t_3_*, *t_4_*: *p* ≤ 0.0000) and increased for all varieties at all harvests when biochar was added (Exps. 1 and 2: *t_1_*, *t_3_*, *t_4_*: *p* ≤ 0.0000). In Exp. 1, at *t_1_*, there was interaction between the varieties and soils (*p* ≤ 0.0017). In Exp. 2, an interaction was observed at *t_1_*, *t_3_*_,_ and *t_4_* (*t_1_*, *t_3_*, *t_4_*: *p* ≤ 0.0000). In both experiments at all sampling times, consistent with the other growth parameters, V3 produced higher fresh biomass, especially when grown with biochar amendment (Figure 2a). Particularly at *t_4_*, plants grown with biochar produced more fresh biomass than controlled soil by 20% and 32% in Exp. 1 and 2, respectively. Dry biomass showed the same trend (Exp. 1: *t_1_*, *t_3_*, *t_4_*: *p* ≤ 0.0000; Exp. 2: *t_1_*, *t_4_*: *p* ≤ 0.0000, *t_3_*, *p* ≤ 0.0425). In both experiments, plants grown in soil with biochar produced more biomass at *t_3_* (Exps. 1 and 2: *p* ≤ 0.0000) and *t_4_* (Exps. 1 and 2: *p* ≤ 0.0000). In Exp. 1, there was an interaction between varieties and soils at the *t_3_* (*p* ≤ 0.0000) and *t_4_* (*p* ≤ 0.0000). In Exp. 2, fresh biomass and dry biomass accumulation were affected by a two-factor interaction at *t_1_* and *t_4_* (*t_1_*: *p* ≤0.0101, *t_4_*: *p* ≤ 0.0000). In both experiments, V3 consistently had the highest dry matter production, especially in soil amended with biochar. Specifically, biochar-treated plants increased dry biomass in both experiments by 50% at *t_4_* compared to non-amended soils (Figure 2b), indicating a potentially beneficial interaction under drought stress.

### 2.2. Root Traits during the Experiments

**Figure 3:**
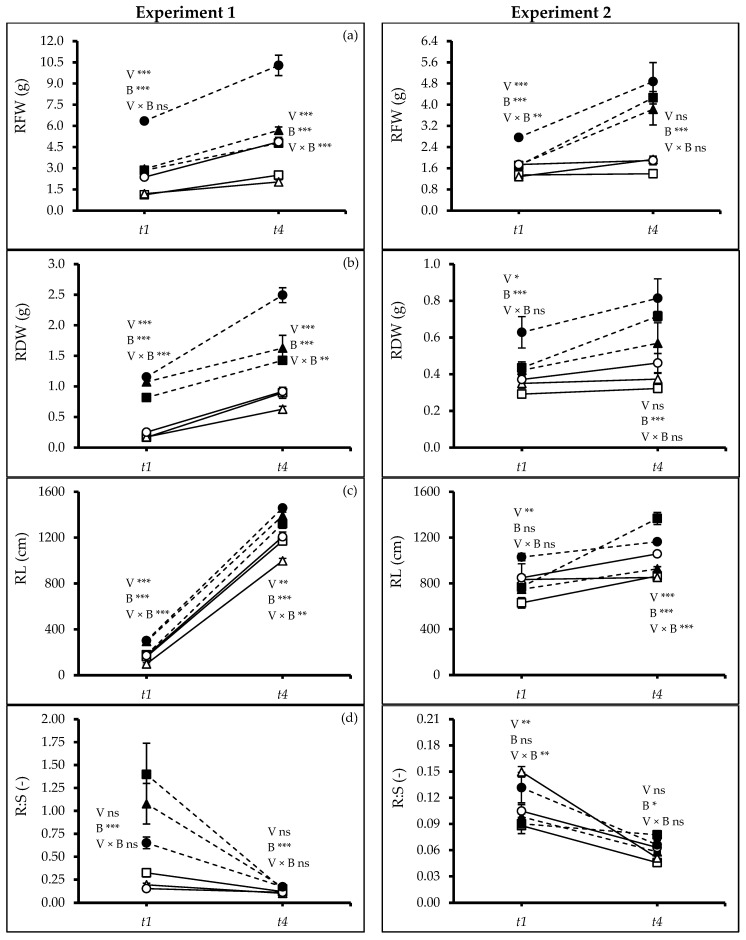
(**a**) Root fresh weight (RFW), (**b**) root dry weight (RDW), (**c**) root length (RL), and (**d**) root shoot ratio (R:S) of quinoa (*Chenopodium quinoa* Willd.) varieties treated without and with woodchip biochar. In each graph, different stars above and below the lines indicate significant differences among treatments (*p* ≤ 0.05, Tukey’s test). F-test significant at *—(*p* ≤ 0.05), **—(*p* ≤ 0.01), ***—(*p* ≤ 0.001). *t_1_*—start of drought, and *t_4_*—end of the experiment. White squares: quinoa variety 1 (V1) without biochar. Black squares: quinoa variety 1 (V1) with biochar; white triangles: quinoa variety 2 (V2) without biochar; black triangles: quinoa variety 2 (V2) with biochar; white circles: quinoa variety 3 (V3) without biochar; black circles: quinoa variety 3 (V3) with biochar; solid lines—(B0) no biochar, dash lines—(B2) 2% biochar.

The root traits varied significantly in both experiments between varieties and growth media, and there was also an interaction. At *t_1_* and *t_4_*, significant differences were observed between varieties, biochar amendment, and interaction for root fresh (RFW) and dry weight (RDW) and root length (RL). Interestingly, plants grown with biochar-amended soils exhibited 130% and 110% more fresh and dry root biomass, respectively, at *t_4_* in both experiments. V3 produced the largest root weight, especially in soil amended with biochar (Exp. 1: *t_1_*: *p* ≤ 0.0000, *t_4_*: *p* ≤ 0.0096; Exp. 2: *t_1_*, *t_4_*: *p* ≤ 0.0000) (Figure 3a–c). In Exp. 1, root shoot ratio (R:S) at *t_1_* and *t_4_* was affected by biochar (*t_1_*, *t_4_*: *p* ≤ 0.0001) but not by varieties. In Exp. 2, R:S varied between varieties at *t_1_* (*p* ≤ 0.0071) and biochar at *t_4_* (*p* ≤ 0.0342). In both experiments, biochar amendment resulted in a higher R:S, probably by enhancing nutrient availability (Figure 3d).

**Figure 4:**
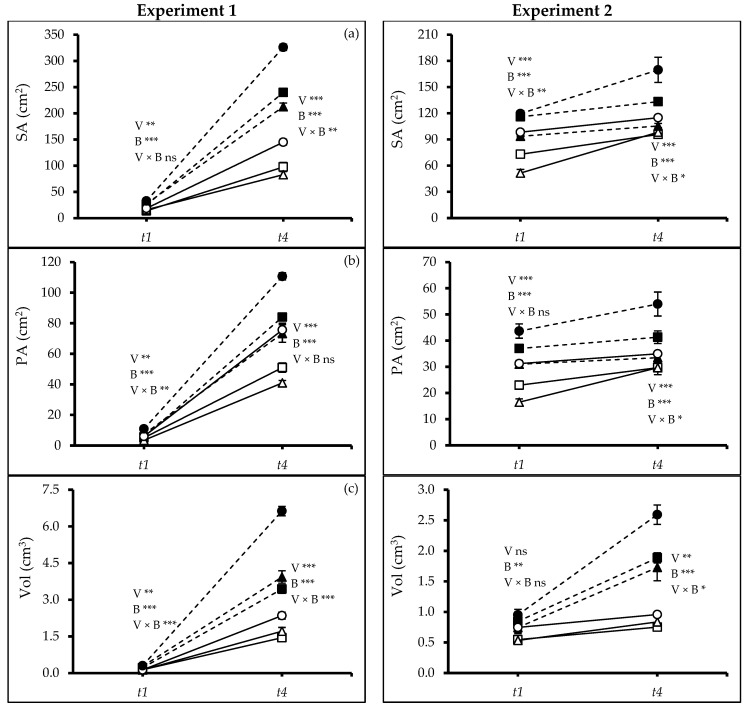

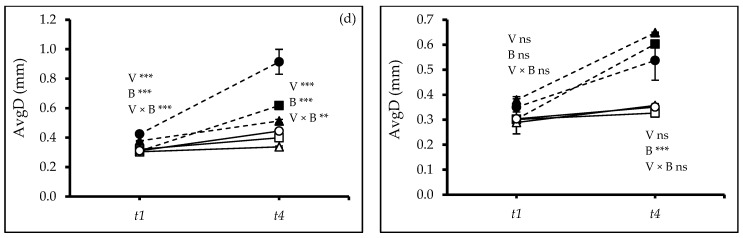
(**a**) Root surface area (SA), (**b**) root projected area (PA), (**c**) root volume (Vol), and (**d**) root average diameter (AvgD) of quinoa (*Chenopodium quinoa* Willd.) varieties treated without and with woodchip biochar. In each graph, different stars above and below the lines indicate significant differences among treatments (*p* ≤ 0.05, Tukey’s test). F-test significant at *—(*p* ≤ 0.05), **—(*p* ≤ 0.01), ***—(*p* ≤ 0.001). *t_1_*—start of drought, and *t_4_*—end of the experiment. White squares: quinoa variety 1 (V1) without biochar. Black squares: quinoa variety 1 (V1) with biochar; white triangles: quinoa variety 2 (V2) without biochar; black triangles: quinoa variety 2 (V2) with biochar; white circles: quinoa variety 3 (V3) without biochar; black circles: quinoa variety 3 (V3) with biochar; solid lines—(B0) no biochar, dash lines—(B2) 2% biochar.

Similarly, root surface (SA) and projected areas (PA) varied in both experiments between varieties (Exps. 1 and 2: *t_1_*, *t_4_*: p ≤ 0.0000) and the two soils (Exps. 1 and 2: *t_1_*, *t_4_*: *p* ≤ 0.0000) both at *t_1_* and *t_4_* (Figure 4a,b). In both experiments, V3 grown in soil amended with biochar had the highest surface and projected root area at *t_1_* and *t_4_*. Root volume and average diameter were different between varieties in Exp. 1 (*t_1_*: *p* ≤ 0.0004, *t_4_*: *p* ≤ 0.0000), soils (*t_1_*, *t_4_*: *p* ≤ 0.0000), and there was an interaction between the two factors (*t_1_*, *t_4_*: *p* ≤ 0.0000). However, in Exp. 2, root volume (Vol) varied between the varieties at t_4_ (t_4_: *p* ≤ 0.0026) but not at *t_1_*. Biochar had a positive effect at both times, i.e., *t_1_* and *t_4_* (*t_1_*: *p* ≤ 0.0091, *t_4_*: *p* ≤ 0.0000). An interaction was also found at *t_4_* (*p* ≤ 0.0292). In Exp. 2, the average diameter (AvgD) varied between soil types at *t_4_* (*p* ≤ 0.0000). In soil with biochar, V3 consistently showed the highest values (Figure 4c,d).

### 2.3. Growth- and Yield-Contributing Traits

**Figure 5:**
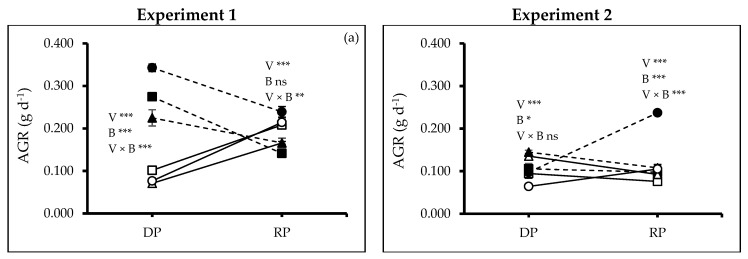

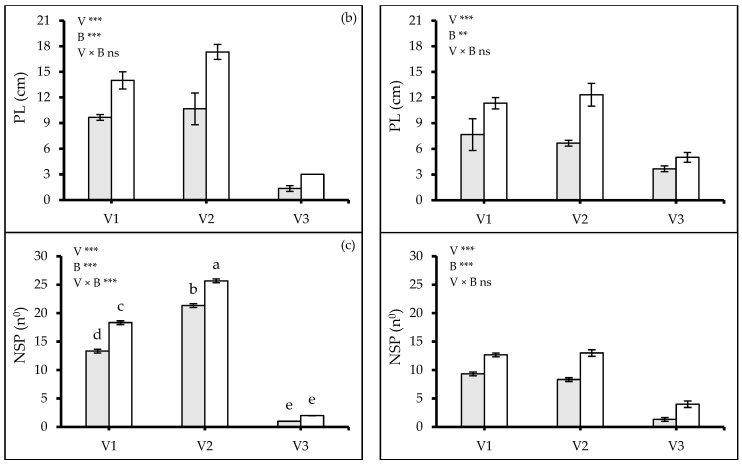
(**a**) Absolute growth rate (AGR), (**b**) panicle length (PL) and (**c**) number of subpanicles (NSP) of quinoa (*Chenopodium quinoa* Willd.) varieties treated without and with woodchip biochar. In each graph, different stars above and below the lines indicate significant differences among treatments (*p* ≤ 0.05, Tukey’s test). F-test significant at *—(*p* ≤ 0.05), **—(*p* ≤ 0.01), ***—(*p* ≤ 0.001). DP—drought period, RP—recovery period. White squares: quinoa variety 1 (V1) without biochar. Black squares: quinoa variety 1 (V1) with biochar; white triangles: quinoa variety 2 (V2) without biochar; black triangles: quinoa variety 2 (V2) with biochar; white circles: quinoa variety 3 (V3) without biochar; black circles: quinoa variety 3 (V3) with biochar; solid lines and grey filled bars—(B0) no biochar, dash lines and unfilled bars—(B2) 2% biochar.

During the drought (DP, between *t_1_* to *t_3_*) and recovery period (RP, between *t_3_* to *t_4_*), the varieties performed differently (Exps. 1 and 2: DP, RP, *p* ≤ 0.0000). In Exp. 1, soil amended with biochar affected the absolute growth rate positively during DP (DP, *p* ≤ 0.0000), but not during RP, while in Exp. 2, biochar had a positive effect during both periods (DP, *p* ≤ 0.0224; RP, *p* ≤ 0.0000). In Exp. 2, there was also interaction between the two factors (DP, *p* ≤ 0.0000, RP, *p* ≤ 0.0046). V3, grown in soil amended with biochar, had the highest growth rate under drought stress (Figure 5a). The varieties also showed different panicle lengths (Exp. 1: *p* ≤ 0.0000; Exp. 2: *p* ≤ 0.0003), which was also positively affected by biochar amendment (Exp. 1: *p* ≤ 0.0002; Exp. 2: *p* ≤ 0.0000). The plants grown with biochar had 57% and 66% longer panicles than controlled soil in both experiments, respectively. V2 had the longest panicles, which became even longer when the plants were grown in soil amended with biochar (Figure 5b). Similarly, during both experiments, the three varieties produced different numbers of sub-panicles (Exp. 1 and 2: *p* ≤ 0.0000), and biochar amendment had a positive effect on the number produced (Exp. 1 and 2: *p* ≤ 0.0000). In Exp. 1, there was also an interaction between the variety and soil (*p* ≤ 0.0000). V2 grown in soil amended with biochar produced the most sub-panicles (Figure 5c).

### 2.4. Physiological and Water-Related Parameters

**Figure 6:**
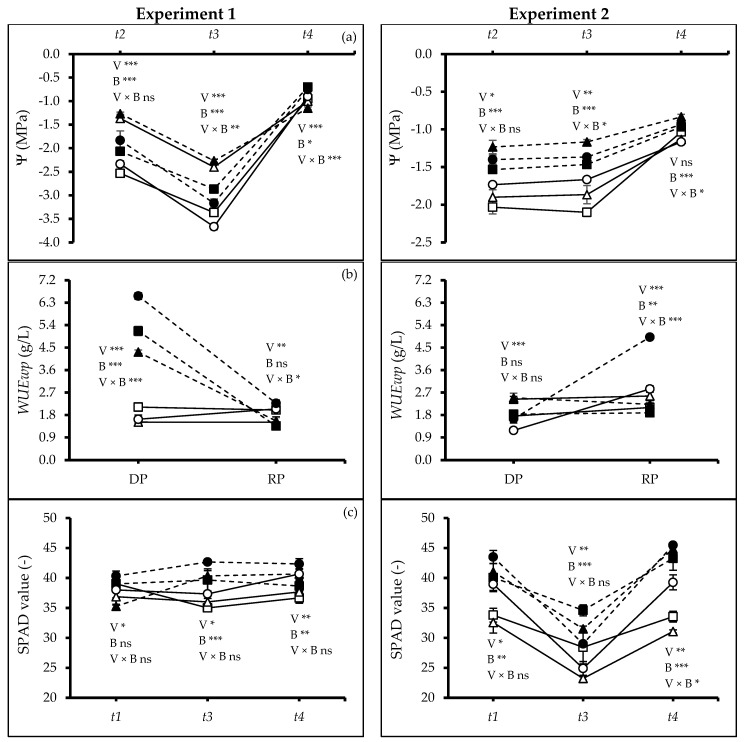
(**a**) Total water potential (Ψ), (**b**) whole plant water use efficiency (*WUEwp*) and (**c**) SPAD index of quinoa (*Chenopodium quinoa* Willd.) varieties treated without and with woodchip biochar. Values are means (*n* = 3) ± S.E. In each graph, different stars above and below the lines indicate significant differences among treatments (*p* ≤ 0.05, Tukey’s test). F-test significant at *—(*p* ≤ 0.05), **—(*p* ≤ 0.01), ***—(*p* ≤ 0.001). *t_1_*—the start of drought, *t_2_*—end of 1st drought cycle, *t_3_*—end of 2nd drought cycle, and *t_4_*—end of the experiment. DP—drought period, RP—recovery period. White squares: quinoa variety 1 (V1) without biochar. Black squares: quinoa variety 1 (V1) with biochar; white triangles: quinoa variety 2 (V2) without biochar; black triangles: quinoa variety 2 (V2) with biochar; white circles: quinoa variety 3 (V3) without biochar; black circles: quinoa variety 3 (V3) with biochar; solid lines—(B0) no biochar, dash lines—(B2) 2% biochar.

In both experiments, the midday leaf water potential (Ψ) was recorded at three different times: (1) at the end of the 1st drought period (*t_2_*), (2) at the end of the 2nd drought period (*t_3_*) and (3) after the recovery period (*t_4_*). At all times, a difference was recorded between varieties (Exp. 1: *t_2_*, *t_3_*, *t_4_*: *p* ≤ 0.0000; Exp. 2: *t_2_*: *p* ≤ 0.0153, *t_3_*: *p* ≤ 0.0011) and soil types (Exp. 1: *t_2_*: *p* ≤ 0.0009, *t_3_*: *p* ≤ 0.0000, *t_4_*: *p* ≤ 0.0228; Exp. 2: *t_2_*, *t_3_*, *t_4_*: *p* ≤ 0.0000), while there were also interactions at *t_3_* (Exp. 1: *p* ≤ 0.0092; Exp. 2: *p* ≤ 0.0141) and *t_4_* (Exp. 1: *p* ≤ 0.0007; Exp. 2: *p* ≤ 0.0383) in both experiments. At *t_2_*, V1 and V3 had lower Ψ values than V2. Biochar amendment significantly improved plant water status. This positive effect of biochar was sustained at *t_3_*, where V1 and V3 in soil without biochar amendment exhibited the most negative values in both experiments (Figure 6a). Following rehydration at *t_4_*, V3 and V1 plants under biochar application recovered leaf water potential more rapidly, suggesting enhanced recovery potential from drought stress.

The total water consumption by plants in Exp. 1 was not significantly different among all experimental factors, while for Exp. 2 the water consumption significantly varied among soil treatment (*p* ≤ 0.0000). Importantly, water consumption by plants treated with biochar was 25% larger than without biochar. Based on this water consumption, *WUEwp* was estimated for two periods: DP (between *t_1_* to *t_3_*) and RP (between *t_3_* to *t_4_*). For DP, in Exp. 1, *WUEwp* varied between varieties (*p* ≤ 0.0000), soil treatments (*p* ≤ 0.0000), and there was also an interaction (*p* ≤ 0.0000). During Exp. 2, the differences were observed among varieties (*p* ≤ 0.0000) but not when they were grown in soil with biochar. More specifically, V3 grown in soil amended with biochar displayed the highest *WUEwp* in both experiments (Figure 6b). For RP in both experiments, a positive effect was observed among varieties (Exp. 1: *p* ≤ 0.0011; Exp. 2: *p* ≤ 0.0000) and also an interaction (Exp. 1: *p* ≤ 0.0139; Exp. 2: *p* ≤ 0.0000). There was only an effect of biochar in Exp. 2 (*p* ≤ 0.0026). V3 grown in soil with biochar maintained the highest *WUEwp* in both experiments (Figure 6b).

The SPAD value (leaf chlorophyll content index) was measured at *t_1_*, *t_3_*, and *t_4_* in both experiments (Figure 6c). At *t_1_*, *t_3_*, and *t_4_*, SPAD values differed between varieties (Exp. 1: *t_1_*: *p* ≤ 0.0146, *t_3_*: *p* ≤ 0.0375, *t_4_*: *p* ≤ 0.0146; Exp. 2: *p* ≤ 0.0218, *t_3_*: *p* ≤ 0.0077, *t_4_*: *p* ≤ 0.0026), however, there was no effect of biochar amendment at *t_1_* but it varied at *t_3_* and *t_4_*, (*t_3_ p* ≤ 0.0000, *t_4_*: *p* ≤ 0.0146) in Exp. 1, while in Exp. 2, it was different at all times (*t_1_*, *t_3_*, *t_4_*: *p* ≤ 0.0000). In Exp. 2, a significant interaction was observed at *t_4_* (*p* ≤ 0.0138). However, in both experiments, V3 had the highest SPAD values, followed by V1. More importantly, control treatment values were significantly reduced, while a sharp increment was observed for biochar-treated plants in both experiments. The same was observed at *t_4_* in Exp. 1 with a difference between varieties (*p* ≤ 0.0025) and soil treatments (*p* ≤ 0.0076), while for Exp. 2, V3 increased SPAD values but it became more pronounced when grown with biochar.

### 2.5. Carbon and Nitrogen Contents

**Figure 7:**
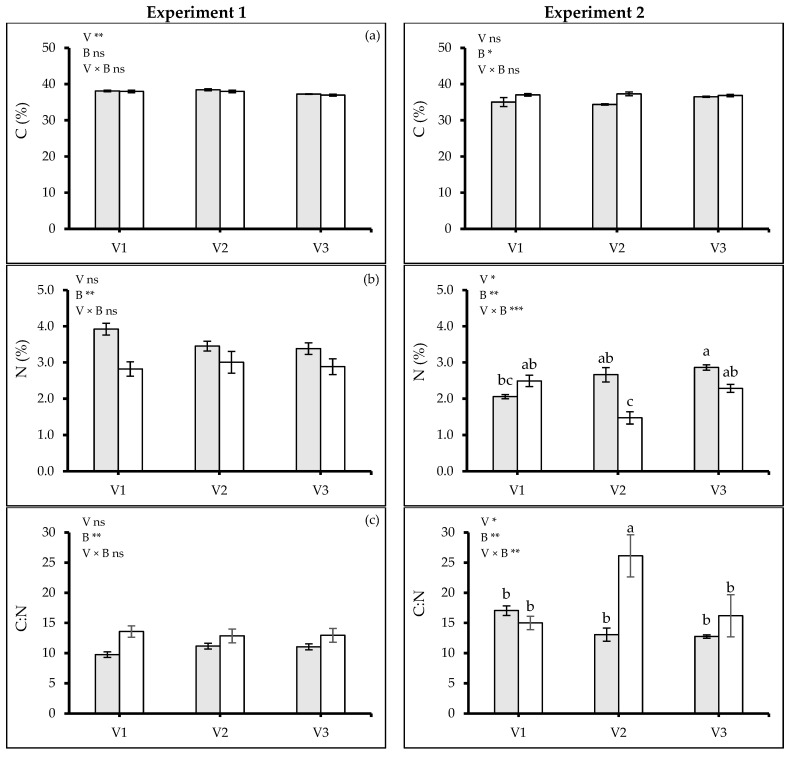
(**a**) Total Carbon% (C, %), (**b**) total Nitrogen% (N, %), (**c**) Carbon–Nitrogen ratio (C:N) of quinoa (*Chenopodium quinoa* Willd.) varieties treated without and with woodchip biochar. Values are means (n = 3) ± S.E. In each graph, different stars indicate significant differences among treatments (*p* ≤ 0.05, Tukey’s test). F-test significant at *—(*p* ≤ 0.05), **—(*p* ≤ 0.01), ***—(*p* ≤ 0.001). V1, V2 and V3—quinoa varieties; grey bars—(B0) no biochar, white bars—(B2) 2% biochar.

At *t_4_*, variations were observed in carbon% (C, %) between varieties in Exp. 1 (*p* ≤ 0.0024) but not in Exp. 2. However, biochar did not have any effect in Exp. 1 but showed variations in Exp. 2 (*p* ≤ 0.0335). The highest carbon% was found in V2 followed by V1 in Exp. 1, while for Exp. 2 plants grown with biochar exhibited higher carbon% (Figure 7a). Biochar had a negative effect on the nitrogen% (N, %) during both experiments (Exp. 1: *p* ≤ 0.0014, Exp. 2: *p* ≤ 0.0018). It did not vary between varieties in Exp. 1 but a significant variation was recorded in Exp. 2 (*p* ≤ 0.0100) (Figure 7b). The results demonstrated that the plants treated with biochar had reduced N in leaves by 21% in both experiments. Plants grown in soil amended with biochar had a greater carbon–nitrogen ratio (C:N) (Exp. 1: *p* ≤ 0.0026, Exp. 2: *p* ≤ 0.0032) (Figure 7c).

### 2.6. Correlation Analysis between SPAD Value and Nitrogen Percentages

**Figure 8:**
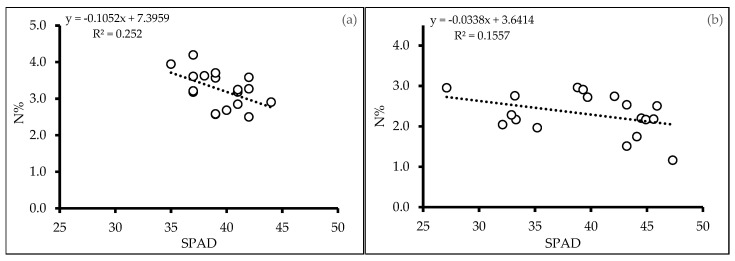
Linear correlation analysis between SPAD values and N content (%) in leaves from experiment 1 (**a**) and experiment 2 (**b**) of quinoa (*Chenopodium quinoa* Willd.) varieties treated without and with woodchip biochar. White circles are measurements.

The observed relationship between SPAD and nitrogen content was weak, as indicated by low R^2^ values of 0.252 and 0.1557, for Exp. 1 and 2, respectively (Figure 8). These findings suggest that while SPAD may be a contributing factor to nitrogen variation, it does not fully explain the observed patterns and highlight the need for further investigation into other factors influencing nitrogen dynamics.

### 2.7. PCA Correlation Analysis at t_4_

**Figure 9:**
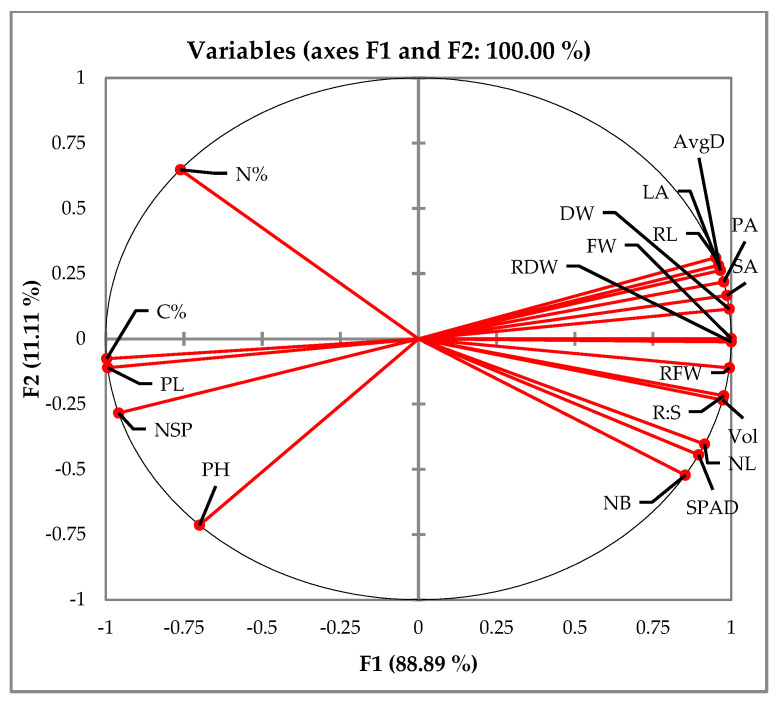
PCA correlation analysis of growth-related attributes at *t_4_* (similar observations were recorded for both experiments). Red arrows indicate the variables included in the PCA, with arrow length representing the contribution of each variable to the principal components. PH—plant height, NSP—number of sub-panicles, PL—panicle length, C%—carbon% in the leaves, N%—nitrogen% in leaves, NB—number of branches, SPAD—SPAD index, NL—number of leaves, Vol—root volume, R:S—root to shoot ratio, RFW—root fresh weight, RDW—root dry weight, FW—fresh weight, DW—dry weight, SA—root surface area, PA—root projected area, AvgD—average density and RL—root length.

A scatter plot was generated to visualize the relationships among plant traits (Figure 9). The PCA suggested potential positive correlations between plant height (PH) and number of leaves (NL), number of sub-panicles (NSP) and panicle length (PL), carbon percentage (C%) and nitrogen percentage (N%), SPAD, and various root parameters (volume, fresh and dry weight, surface and projected area, and length). Conversely, a potential negative correlation was observed between root to shoot ratio (R:S) and both fresh weight (FW) and dry weight (DW).

## 3. Discussion

### 3.1. Influence of Biochar Application on Growth under Drought Conditions

The woodchip biochar application significantly enhanced growth under water stress during the vegetative phase for all varieties but especially for the drought-resistant variety UAFQ7 (V3) (Figure 1 and Figure 2). The drought-sensitive variety *Titicaca* was more negatively affected by prolonged water shortage than the others even when it grew in soil amended with biochar [19].

The experimental results showed significant differences between varieties in terms of plant height, number of leaves and branches, and leaf area when grown with and without biochar amendment (Figure 1 and Figure 2), which is in line with previous studies [19,23,31]. The drought-sensitive variety Titicaca produced more biomass when it grew in the biochar-amended soil as previously observed [19,23]. Panicle numbers and length are early determinants of grain yield [32], and water stress affected these parameters negatively [33]. Our results confirmed that the application of biochar as soil amendment offers a promising mitigation strategy, as shown before [34]. García-Parra et al. [35] reported that quinoa’s developmental stages influenced the panicle formation and other yield components [36]. The faster vegetative growth of UAFQ7 results in increasing production of leaves and branches, and a larger biomass. However, the enhanced vegetative growth came at the expense of panicle formation [37]. Some UAFQ7 plants even exhibit shorter panicles or lack sub-panicles entirely (Figure 5b,c). This suggests a potential trade-off between vegetative growth and panicle development under biochar application during water limitations.

### 3.2. Influence of Biochar Application on Physiology and Water-Related Traits under Drought Conditions

Our study revealed that biochar addition to the soil significantly improved the plants’ water status by making the midday leaf water potential less negative than plants grown in unamended soil (Figure 6a). Some studies found that quinoa initiates stomatal closure when leaf water potential falls below −1.2 to −1.6 MPa [38,39]. Accordingly, the stomata might have been closed at the end of drought stress in the present study as the leaf water potential had dropped to below −2.0 MPa (experiment 1) and −1.5 (experiment 2), particularly for those grown in soil without biochar amendment. The lower water potential could be due to the intrinsic lower osmotic potential of the plants [7]. The osmolyte accumulation contributes to lowering the water potential and enhancing root growth in quinoa [7,33].

Our findings support the hypothesis that biochar application helps plants to maintain water status under drought. Gaskin et al. [40] reported an increased accumulation of osmotically active substances like potassium (K^+^) in plant tissues when biochar was added to the soil. The high cation content in biochar may facilitate improved plant water uptake and higher water use efficiency. Besides, drought tolerance in quinoa has also been linked to dense root elongation supported by our study (Figure 3 and Figure 4) and the presence of hygroscopic calcium oxalate crystals in leaf cuticles, which can further minimize water loss [41]. Issa Ali et al. [42] observed the same for Titicaca and L119 varieties.

Biochar application improved SPAD values in quinoa varieties under water stress (Figure 6c). The increase in SPAD could be caused by the large surface area of biochar, facilitating enhanced adhesion and cohesion with water and increased soil acidity and subsequent heightened production of photosynthetic pigments. Anee et al. [43] also found an increased SPAD index of wheat when plants were grown in soil amended with biochar. However, the increased SPAD value was seemingly not associated with the changes in leaf nitrogen content, as the latter was negatively affected by biochar amendment (Figure 7b).

### 3.3. Influence of Biochar Application on Root Morphology and Leaf Nutrient Concentrations under Drought Stress

Biochar application stimulated the root growth resulting in a deeper root system (Figure 3 and Figure 4), which could further enhance water acquisition through the finer pores within the biochar itself, reducing the negative leaf water potential and subsequently improving plant water use efficiency (Figure 6a,b). Jabborova et al. [44] reported significant improvement in the root morphology of okra under woody biochar applications likely caused by an enhanced nutrient acquisition [45] and potentially improving the soil microbial community [46]. Biochar amendments significantly enhanced the leaf’s relative water content, osmotic potential, photosynthetic rate, and WUE reflected in higher SPAD values (Figure 6c). Similar results were found for maize cob biochar [26] and woody biochar [18] on quinoa and crops like rice [47], wheat [48], maize [49], soybean [50], and tomato [51]. Our findings on increased root-to-shoot ratio and absolute growth rate under woodchip biochar application (Figure 3d and Figure 5a) align with previous studies [52,53]. The woodchip biochar has probably increased the nutrient absorption capacity of the quinoa roots. Additionally, biochar application may contribute to better plant health by accelerating systemic defenses and reducing the soil pathogen load [45]. Collectively, these mechanisms likely contribute to the observed improvement in plant growth efficiency under biochar application. Furthermore, woodchip biochar addition can increase soil organic carbon, potentially leading to a higher carbon-nitrogen ratio (Figure 7), as reported by Zhao et al. [54]. This can enhance soil nitrification and improve nitrogen bioavailability, ultimately promoting plant biomass accumulation (Figure 2).

## 4. Materials and Methods

### 4.1. Plant Material and Growing Conditions

Two identical experiments were conducted under controlled greenhouse conditions at the Department of Plant and Environmental Sciences, University of Copenhagen, Taastrup, Denmark (55.66 N-12.30 E, 27 m a.s.l.). The first experiment (Exp. 1) was conducted from October 2023 to January 2024, and the other (Exp. 2) from January to March 2024. A completely randomized factorial design with nine replicates was employed, with the three varieties Titicaca (V1), Quipu (V2), and UAFQ7 (V3) (Table 1) grown in soil without (B0) and with biochar (B1) resulting in a total of six treatments, i.e., V1B0, V2B0, V3B0, V1B2, V2B2, V3B2.

In each experiment, nine plastic pots (diameter: 26 cm; height: 20 cm; volume: 7.5 L) were filled with sandy loam soil collected from the Taastrup campus fields and nine were filled with sandy loam soil mixed with woodchip biochar manufactured by Nerabiochar (Nerabiochar, Ivrea, Torino, Italy). The properties of the biochar are shown in Table 2 The biochar was incorporated into the soil (Table 3) at the rate of 2% based on the dry weight of the soil. Soil moisture was estimated (Equation (1)) after weighing the soil before (*FW*) and after drying in a drying chamber for two days at 105 °C (*DW*). The pots were placed in a greenhouse with 16/8 h light/darkness, at a temperature of 20 ± 2 °C, and a relative humidity of 70 ± 7%.

(1) $$\mathrm{Soil}\;\mathrm{water}\;\mathrm{content}\left(\%\right)=\frac{\left(FW-DW\right)}{\left(DW\right)}\times 100$$

Similarly, the soil properties used in the experiment are listed in Table 3.

Ten seeds per pot were sown. When plants had 2−4 leaves, the number of plants was reduced to three uniform plants. At the 6-leaf stage, the plants were thinned to one plant per pot. From emergence to the 12-leaf stage, the plants were kept well-watered. At the 12-leaf stage, two successive drought cycles were imposed.

### 4.2. Water Stress Treatment

From emergence to the 12-leaf stage, pots were regularly watered to 100% pot water holding capacity (WHC) when soil water content reached 70% of WHC, determined by daily weighing the pots between 8:00−9:00 AM. The 100% pot WHC was defined as pot weight when drainage had stopped after saturation of the soil. At the 12-leaf stage, two progressive drought cycles were applied, one after the other. In both experiments, the stress period started after the water content in all pots was brought to 100% pot WHC at 35 Days After Sowing (DAS) (time = *t_1_*). At *t_1_*, three replications were harvested at the soil surface, and plant height, number of leaves and branches, leaf area, and fresh and dry biomass of the above-ground plants were recorded. The watering of the remaining pots was retained until all available water was used at 45 DAS (time *t_2_*). Then, plants were rewatered to field capacity, and the second drought period began. It finished at 56 DAS (time = *t_3_*). At *t_3_,* three replications were harvested, and their yield components were recorded. The remaining three replications kept growing under well-watered conditions until flowering initiation happened 80 DAS (Time = *t_4_*). Then, plants were harvested, and their yield components were measured.

### 4.3. Measurements

#### 4.3.1. Morphological Parameters

Plant height (cm), number of leaves (n^0^), and branches (n^0^) were recorded at times *t_1_*, *t_3_*, and *t_4_* by sampling three plants. The fresh weight (g) of above-ground biomass (*TFW*) was measured at each time. Afterwards, the plants were dried in an oven at 70 °C until constant weight and the dry weights were recorded. Yield components such as main panicle length (cm) and the number of sub-panicles plant^−1^ were determined at *t_4_*. At each harvest point (*t_1_*, *t_3_*, *t_4_*), the leaves were collected, and the leaf area (LA_1_, LA_3_, LA_4_) was determined by a leaf area measuring instrument (model 3100; LI-COR, Inc., Lincoln, NE, USA).

Finally, the Absolute Growth Rate (*AGR*) (Equation (2)) of the plants was calculated for the drought period (DP, between *t_1_* to *t_3_*) by recording the plant dry weight at the start (*DW_1_*) and end of the drought treatment (*DW_2_*):

(2) $$AGR(g\;d-1)=\frac{\left({DW}_{2}-{DW}_{1}\right)}{\left(t_{3}-t_{1}\right)}$$

Likewise, *AGR* was calculated for the recovery period (RP, between *t_3_* to *t_4_*) using *DW_3_* and *DW_2_*.

#### 4.3.2. Root Measurements

Root fresh and dry weight (g), and root shoot ratio (R:S) were estimated at *t_1_* and *t_4_* from 3 plants. Additionally, at the same time, roots were scanned in a root scanner by using Epson scan 2 software (https://epson.com/Support/Scanners/Expression-Series/Epson-Expression-12000XL---Photo/s/SPT_12000XL-PH?review-filter=Windows+11, accessed on 14 August 2024), and root length (cm), projected area (cm^2^), surface area (cm^2^), volume (cm^3^), and average diameter (mm) were measured via WinRhizo software 7.6.5.

#### 4.3.3. Water-Related Parameters

At the end of the drought periods (*t_3_*) and the trials (*t_4_*), the total water consumption (*TWC*) was calculated by cumulating the amount of water consumed by the plants. Water consumption was measured by weighing the pots daily. Then, the whole plant water use efficiency (*WUEwp*) during the drought period was calculated according to Equation (3) [55]:

(3) $$\mathrm{WUEwp}=\frac{{\mathrm{DW}}_{2}-{\mathrm{DW}}_{1}}{\mathrm{TWC}}$$

where *TWC* is the total water consumed during the drought period. Likewise, *WUEwp* was calculated for the recovery period using *DW*_2_, *DW*_3_ and *TWC*. Midday leaf water potential (Ψ) was measured in a pressure chamber (Soil Moisture Equipment Corp., Santa Barbara, CA, USA) on three fully expanded upper canopy leaves between 12:00 and 13:00 h at *t_2_*, *t*_3_ and *t*_4_. For that purpose, the young fully expanded leaf was detached from the plant along with its petiole. The leaf was then placed in the chamber with the petiole protruding outside. Afterwards, the pressure was increased gradually in the chamber and the reading was recorded immediately when a droplet of water came out from the protruding petiole surface.

#### 4.3.4. Measurements of SPAD Index

Relative leaf chlorophyll content was assessed using a chlorophyll meter (SPAD-502; Konica Minolta Sensing, Inc., Osaka, Japan). Five measurements were recorded on five fully expanded top leaves of each plant, and the values were subsequently averaged.

#### 4.3.5. Total Leaf Nitrogen and Carbon Contents

Three dried leaf samples were collected at *t_4_* and finely ground. The total leaf N, C, and H content (% *DW*) were estimated with a CHNS/O elemental analyzer (Flash 2000, Thermo Fisher Scientific, Cambridge, UK) using the dynamic flash combustion technique known as the modified Dumas method [56].

### 4.4. Statistical Analysis

A two-way analysis of variance was computed using the statistical software RStudio 4.2.0 [57], considering varieties and biochar levels as the main factors. Mean comparisons among treatment groups were performed using Tukey’s Honestly Significant Difference (HSD) test at a significance level of *p* ≤ 0.05. Similarly, correlation analysis between SPAD values and N% in the leaves and principal component analysis (PCA) was done by using the same software.

## 5. Conclusions

Biochar application improved plant water status, growth, and root development. The positive effect of woodchip biochar is likely caused by influencing several mechanisms like improved water uptake, soil features, and enhanced nutrient availability. However, biochar application may lead to a trade-off between vegetative growth and panicle development under water stress in some quinoa varieties. Overall, this study suggests that a 2% application of woodchip biochar is a promising strategy for enhancing drought tolerance and growth of quinoa, although the positive effect varies between varieties (Titicaca < Quipu < UAFQ7). As these varieties responded differently to water stress conditions, so, further investigation is needed to elucidate the underlying mechanisms by which biochar influences stomatal behavior and drought response among different quinoa varieties

## Figures and Tables

**Table 1:** Varieties used in the experiments.

Varieties	Code	Origin	Collection Site/Company
Titicaca	V1	Denmark	University of Copenhagen, Taastrup, Denmark
Quipu	V2	Italy	Tuttoquinoa di Vannuzzi Dario, Manciano, Italy
UAFQ7	V3	Pakistan	Muhammad Nawaz Shareef University of Agriculture (MNSUAM), Multan, Pakistan

**Table 2:** Woodchip biochar amendment properties used in the experiment (from [23]).

Parameters	Biochar
pH	8.9 ± 0.13
EC	0.52 ± 0.04 dS m^−1^
Moisture	5.6 ± 0.11% dw
Volatile solids	42.3 ± 0.44% dw
Ash	4.4 ± 0.21% dw
Fixed carbon	53.3 ± 0.24% dw
C	68.3 ± 0.11% dw
H	4 ± 0.04% dw
N	1 ± 0.03% dw
C_org_	66.3 ± 0.06% dw
C/N	67.2 ± 1.96
S	0.03 ± 0.01% dw
O	22.3 ± 0.29% dw
H/C_org_ ratio	0.06 ± 0.01
O/C_org_ ratio	0.4 ± 0.01

Values are means (*n* = 3) ± s.e. dw = dry weight

**Table 3:** Soil properties used in the experiment.

Parameters	Units	Soil
Phosphorus	(mg/100 g)	4.7
Potassium	(mg/100 g)	19
Magnesium	(mg/100 g)	5.9
Sodium	(mg/100 g)	2.6
Manganese	(mg/kg)	30.2
Copper	(mg/kg)	1.5
Zinc total	(mg/kg)	2.4
Boron	(mg/10 kg)	3.4
Molybdenum	(mg/10 kg)	0.92
Organic matter	(%)	2.14
Clay (<0.002 mm)	(%)	9.1
Silt (0.002 mm)	(%)	7.0
Fine sand (0.02–0.2 mm)	(%)	43.5
Coarse sand (0.2–2.0 mm)	(%)	38.4

## Data Availability

The data presented in this study are available upon request from the corresponding authors.
